# Supplementary material for: Are carbon emissions trading and green financial instruments synergistic? -Comprehensive quantitative research based on content analysis
Source: PLoS One. 2024 Mar 7;19(3):e0298601. doi: 10.1371/journal.pone.0298601 (PMC10919601; doi:10.1371/journal.pone.0298601)
Supplement: S1 Data — (ZIP) [file pone.0298601.s001.zip › Data/地级市Z_F工作报告.zip!/╔╜╢1⁄2/╬1⁄2╖╗╩╨/└┤╫╘╣1⁄2╓┌║┼í╛┬φ┐╦╩2╛▌═°í┐_2014.docx]

各位代表:

现在,我代表市政府向大会报告工作,请予审议,并请市政协委员和其他列席会议的同志提出意见。

一、2013年工作回顾

2013年,在中共潍坊市委的坚强领导下,在市人大及其常委会、市政协及社会各界的监督支持下,市政府与全市人民一道,紧紧围绕“四一三”战略重点和全市统筹发展战略布局,改革创新,开拓进取,扎实工作,圆满完成了市十六届人大二次会议确定的目标任务。

经济持续健康发展。全市实现地区生产总值4420.7亿元,增长10.6%。完成公共财政预算收入383.9亿元,增长15.6%。完成固定资产投资3430亿元,增长19.5%。实现社会消费品零售总额1758.8亿元,增长13.4%。城镇居民人均可支配收入、农民人均纯收入分别达到28386元、13273元,增长10%和12.5%。居民消费价格涨幅控制在1.6%。

产业转型深入推进。以建设“四个基地”为重点,大力推动产业转型。围绕发展品牌农业进行转调创。新增现代农业园区110个,登记家庭农场1808家,新增农民合作社2695家,“三品一标”总数达到1636个。农业部将我市确定为国家现代蔬菜种业创新创业基地和农业国际合作示范区。深入实施工业提升计划,着力加强“五项建设”,13个专项计划相继出台,完成工业技改投资1160.2亿元。规模以上工业主营业务收入、利税分别增长13.5%和22.8%。潍柴集团主营业务收入突破一千亿元,歌尔声学市值突破530亿元。高新技术、新兴产业、非加工制造环节产值占规模以上工业总产值比重均提高1个百分点以上。新成立国家级产业技术创新战略联盟3家。潍柴动力国家工程技术研究中心通过验收,盛瑞传动国家工程技术研究中心启动建设,首条8AT生产线顺利投产。服务业提速计划顺利实施,增加值占比提高1.95个百分点,高于全省0.75个百分点。总部经济、金融、现代物流、商贸服务、健康养老等产业加快发展。健康产业规划、文化艺术品交易中心规划、“金创33条”等相继出台。银行机构存贷款余额分别突破5000亿元、4000亿元,增量居全省第5位和第3位。投资100亿元以上的寒亭总部基地、汉富集团食品谷总部基地正式签约。全市首个5A级旅游景区获批。企业主辅业务分离成效显著,新增限额以上服务业企业2151家。

统筹发展格局更加完善。举全市之力突破滨海,海洋经济新区建设规划基本编制完成,15个飞地经济产业园开工建设。潍坊港3个万吨级泊位正式对外开放,3个两万吨级泊位新投入使用,5万吨级航道和码头获批并开工建设。鲁辽陆海货滚甩挂运输通道项目加紧论证。潍日高速开工建设,新机场选址已经确定,济青中线前期工作启动,高铁枢纽列入国家规划。边调研边实施,着力提升市区。《全面深化改革大力提升市区科学发展水平的决定》已经形成并将择机出台。理顺了市区财政体制,一批审批管理权限及时下放。提升市区各专项规划加快编制。中国食品谷、测绘地理信息产业基地等重点片区、园区建设顺利推进。市区公共自行车系统投入运营。一批提升市区的重点项目纳入计划。开展两河开发战略性研究,形成了初步成果。新型城镇化体系建设加快推进。《潍坊市城镇化发展规划》编制完成,区域重点镇试点扎实开展,新增财政收入过亿元镇5个。规划农村社区1182处,建成965处。全市城镇化率提高2.01个百分点,达到51.8%。

改革开放步伐加快。在全省率先完成农村集体建设用地和农村宅基地确权登记颁证,60%的村庄完成了土地承包经营权确权登记颁证。面向全省的齐鲁农村产权交易中心获批建设。工商登记制度改革成效显著,下半年新增市场主体4.1万户,环比增长49.8%。原有16个市级政府融资平台顺利整合为8个规范化公司。在全省率先成立了地方金融监管局和金融控股集团。中高职与普通本科人才实现衔接培养。出台发展民办教育10条措施,吸引社会投资53亿元。公立医院改革试点顺利完成。完成进出口总额162亿美元。综合保税区一期封关运行。香港山东周、“莫言家乡·魅力潍坊”宝岛行等系列经贸交流活动成功举办。与德国弗莱辛地区结为全国首批中欧城镇化伙伴关系城市。

生态文明建设扎实推进。全面启动“386”环保行动,实施4个月来,关停污染企业581家,拒批化工类项目63个,对4个区域实施了限批。城区“蓝天白云、繁星闪烁”天数居全省第7位,23条重点河流水质Ⅴ类以上断面提高7个百分点,化学需氧量、氨氮、二氧化硫、氮氧化物排放总量降幅均超过前两年总和。实施绿色建筑行动,新开工建设低碳社区414万平方米。万元GDP能耗降低3.8%。连续14年实现耕地总量动态平衡。完成绿化造林34.96万亩。

民生和社会事业持续改善。民生支出占公共财政支出比重达到58.2%,比上年提高2.1个百分点。市政府承诺的25件民生实事全面完成。深入开展全国文明城市创建活动,创建为首批省公共文化服务体系示范区和首届文化强省建设先进市。城市文明指数测评居全国同类城市前列。圆满完成十艺节承办任务并取得优异成绩。被授予“中国画都”称号。新改扩建普惠性标准化幼儿园289所,740所农村小学全部达到标准化办学条件。新增城镇就业12.5万人,转移农村劳动力12.4万人。市大学生创业孵化中心投入使用。强化基本民生保障,提高了全市最低工资标准、低保标准和五保供养标准。养老事业、养老产业协调发展,新增养老床位9600张。阳光保险与市人民医院、潍坊医学院合作运营市民健康中心项目正式签约。市级心理健康服务中心挂牌成立。农村饮用水率先整建制达到国家标准。平安潍坊、法治潍坊建设深入推进,安全生产主要指标继续下降,群众安全感测评列全省第1位。双拥共建、优抚安置、人口计生、妇女儿童、老龄助残、社会救助等继续取得优异成绩,民族宗教、外事侨务、经济普查、对口支援等工作实现新发展。

政府自身建设进一步加强。持续推进责任政府、法治政府、效能政府、创新政府、廉洁政府建设。严格执行市人大及其常委会决议决定,自觉把民主政治协商纳入决策程序,主动听取社会各界意见。共办理人大代表议案建议163件、政协委员提案324件。推行政府重大决策和经济合同合法性、合理性、风险性评估审查制度、行政裁量权基准制度。按照“八最”要求,深化行政审批制度改革,压减市级审批事项45.3%。市级行政事业性收费由118项减少为56项,减轻企业和社会负担2.2亿元。市级3305项行政处罚事项纳入在线监控。查处损害发展环境案件640起,处理相关责任人1966人。开展中介组织领域突出问题专项治理,查办违纪违法案件37起。认真执行中央“八项规定”,公务接待、办公用房等得到较好规范和治理。

过去一年的成绩来之不易。这是省委、省政府和中共潍坊市委正确领导的结果,是市人大及其常委会、市政协和社会各界有效监督、大力支持的结果,是全市广大干部群众团结一心、拼搏进取的结果。在此,我代表市政府,向所有关心、支持、参与潍坊改革发展并作出贡献的各级领导、上级各部门、社会各界、驻潍解放军和武警官兵、海内外朋友,表示衷心的感谢!

同时,我们也清醒地看到,经济社会发展还存在不少问题和压力,主要是:人均水平不够高,城乡之间、区域之间发展不够平衡；发展方式比较粗放,经济结构和质量效益亟待优化提升；资源环境压力日趋加大；发展活力、创新能力亟待增强；民生保障离群众要求还有较大差距,部分群众生活还比较困难；政府职能转变、社会治理体系建设、工作人员职业化建设滞后于形势发展要求；腐败和损害群众利益的现象还时有发生。对此,我们要高度重视,采取有效措施加以解决。

二、2014年政府工作总体要求和目标任务

今年是全面深化改革的第一年,是全面完成“十二五”规划的关键一年。我们既面临一系列挑战,又面临难得的发展机遇。做好今年的工作,既要强化底线思维,有效预防和管控好各种风险；又要强化辩证思维和竞争意识,善于发现和抓住各种发展机遇,坚定自信心,汇聚正能量,满怀豪情地开创全市科学发展的新局面。

今年的政府工作,要认真贯彻落实中央和省委、省政府决策部署,牢牢把握稳中求进、改革创新的核心要求,以提高发展质量和效益为中心,加快转变发展方式,加快转变政府职能,加快转变工作作风,着力推动产业转型,着力激发市场活力,着力保障和改善民生,促进经济持续健康发展和社会和谐稳定,努力实现更高层次新发展,加快建设富裕文明新潍坊。

今年,全市经济社会发展的主要目标为:地区生产总值增长9.5%,公共财政预算收入增长12%,固定资产投资增长17%,社会消费品零售总额增长13.5%,进出口总额增长6%,城镇居民人均可支配收入和农民人均纯收入均增长10.5%,城镇登记失业率控制在3.8%以内,居民消费价格涨幅控制在3.5%以内。人口自然增长率控制在8.95‰以内。节能减排目标在省下达指标基础上加严15%。

以上目标,是综合考虑今年经济形势、“十二五”规划,特别是实现工作指导重大转变的要求提出的。节能减排、人口增长为约束性指标,必须完成；其他均为预期性指标,要从实际出发,不盲目攀比,不层层加码,不简单以GDP增长率论英雄；把精力更多放到腾笼换鸟、转型发展上；放到深化改革、创新驱动上；放到更好地保障改善民生和促进社会和谐上。在保证质量效益的前提下,争取更为合理的增长速度,实现更高质量的发展。为此,今年增加了一系列体现转型发展要求的指标,提请大会一并审议。

根据以上要求和目标,今年重点做好七个方面工作:

(一)加快推进产业转型,在构建现代产业体系上实现新突破。立足实际,面向未来,以加快建设“四个基地”为目标,推动产业向高端高质高效方向转型发展。

——服务业特别是现代服务业,是提升地区竞争力的关键。要继续大力实施服务业提速计划,完善机制,创新业态,促进服务业发展提速、比重提高、质量提升。突出抓好总部经济、创意设计、现代物流、金融服务、健康养老、休闲旅游、信息消费、服务外包、社区服务等重点产业,规划建设好特色服务业聚集区,培育引进行业龙头企业和骨干企业,打造完整产业链。

总部经济高度集约、高效产出,要优先发展。加快寒亭总部基地、食品谷总部基地、文化产业总部基地等建设步伐,鼓励各县市区、开发区和市直有关部门创办、领办各类特色总部基地,继续大力培育引进各类总部及分支机构,力争年内新增各类总部机构100家以上。

创意设计产业是创新的源头,我市资源丰富,发展前景广阔。要推动创意设计与产业、城市融合发展,加快打造为战略性支柱产业。全面提升潍坊软件园、潍坊广告创意产业园、潍坊文化产业孵化器管理体制和运营水平。加快实施潍坊文化艺术品交易中心规划,创办市文化创意产业园、工业设计中心、文化创意学院。加快推进160个投资过亿元文化产业项目建设。

现代物流是企业的“第三利润源”,是提质增效的“助推器”。要加快编制现代物流基地发展规划,统筹推进临港物流、空港物流、保税物流、食品谷物流和鲁东物流等物流园区建设,提升大宗商品交易市场发展水平。推动线上线下一体化发展,提升物流业信息化、专业化、现代化水平,打造现代物流基地。年内物流业总额增长20%以上。

金融业既是经济社会健康运行的保障,也是战略性产业。要抢抓机遇,创新发展,全力打造。全面落实“金创33条”,加快扩张主体、开拓市场、优化结构、创新业态,形成金融与产业、科技、文化等各领域深度融合发展的体制机制。加快建立职业化的地方金融监管体系,完善属地管理与行业管理并重的金融风险管控责任制和工作机制。年内,以县为单位,建立起规范的民间借贷管理服务中心。

制造服务化是传统制造业转型升级的方向,也是加快服务业发展的最大潜力,要作为重大专项谋划推进。组织开展好专项培训辅导,强化政策支持和服务保障,年内争取5%左右的规模以上制造企业能够开展整体解决方案、系统集成与工程总承包、融资租赁、供应链管理、再制造等服务业务。今年全市服务业占比力争再提高2个百分点。

——高新技术及战略性新兴产业(以下简称新兴产业)是未来发展的战略制高点,是增强我市产业竞争力的关键所在。要坚持有所为、有所不为,精心实施好新兴产业培育工程,集中力量、集成要素,加快培育,力争高新技术产业占比每年提高2个百分点左右。突出蔬菜种业优势,依托骨干企业和园区,加大扶持力度,深化国际合作,把我市打造成国内蔬菜种业发展的先行区。着眼于我市基础优势和未来竞争需要,集中培强做大电子信息、生物医药、节能环保、智能装备、新能源及电动汽车等新兴产业,力争3-5年内,成为全市支柱性产业。深入实施开发区转型计划,加快体制、管理、服务创新步伐,进一步发挥各级开发区、各类特色产业园区特别是高新区在新兴产业培育中的引领、示范、带动作用。鼓励境内外机构、大企业特别是民营资本参与、领办各类园区。支持高新区按照全市总体规划,在全市范围内布局发展特色新兴产业园区,在境内外布局创新孵化器。大力培育高新技术企业,形成上下游衔接、大中小配套的高新技术产业集群。

——机械装备、汽车制造、石化盐化、纺织服装、食品加工、造纸包装等优势传统产业,是我市的当家产业,也是我市产业转型的重点和关键。只有被淘汰的企业,没有被淘汰的产业。传统优势产业加快转型,将成为我市竞争力的重要支撑。继续大力实施工业提升计划,选择重点产业,搞好专业诊断咨询,进一步明确方向、落实关键措施。以企业转型支撑产业转型。依据产业标准和市场状况,搞好企业分类指导和服务,鼓励发展一批,改造提升一批,优化重组一批,转产迁出一批,关闭淘汰一批。上级下达的淘汰落后产能任务要不折不扣完成。以信息化引领工业现代化,深入推进“两化”融合工程。实施好700个投资过亿元技改项目。大力推动产品升级。每个产业选择30个左右的重点企业,推动其主导产品由中间产品向终端产品延伸,由低端产品向高端产品、品牌产品延伸,由单一功能产品向智能化、多功能产品延伸,由一般性产品向进口替代产品、产业链短缺产品和创意产品延伸,使传统优势产业进一步焕发生机和活力。

——农业是民生之本、稳定之要、发展之基。要把重中之重的要求落到实处,围绕“三个导向”,以优质高效生态安全为主题,以品牌农业为引领,加快建设现代农业示范基地。大力实施耕地质量提升计划和粮食百亿斤工程。有序推进农业规模化经营,加快农村土地向各类新型经营主体集中。年内,力争新增各类农民合作社500家、家庭农场2000家。因地制宜发展一批现代农业园区,持续提升生态农业、信息农业、都市农业、休闲观光农业等现代农业比重。依托各类新型经营主体,大力培育农产品品牌尤其是知名品牌。大力推广出口农产品质量管理模式,加快建立等级分明、标准明晰、可追溯、可查询、全覆盖的食品、农产品质量管理体系,整建制创建农产品质量安全区。按照零容忍、规范化、全覆盖的要求,建立高效、权威、一体化的食品药品安全监管体系,强化社会监督、舆论监督和法律监督,依法严厉打击各种违法行为和渎职行为。

加快培育现代产业体系。充分发挥市场主导、企业主体和政府引导作用,促进各类要素向重点产业高效聚集,加快形成市场前景好、产业聚集度高、有较强竞争力的现代产业体系。实施现代产业培育工程,围绕种苗产业、5个战略性新兴产业、9个重点服务业和部分传统优势产业,逐一完善规划,落实重点服务部门和专业招商服务团队,规划建设好重点园区,建好产业基金,加快建立起完善的产业发展服务体系,促进传统产业加快转型升级,新兴产业加快培育壮大。把项目作为培育产业、加快转型的重要抓手,全力抓好一批大项目、好项目。建立项目目标责任制,领导干部亲自抓,各级各部门共同抓。已签约的项目,要加快落地,争取及早投产运营；有意向的项目,要盯紧靠牢,力争及早签约。进一步健全完善招商体制机制,形成招商促进转调创的新格局。坚持企业主体地位,支持潍柴等大企业继续做大做强,扶持中小企业进一步做精做优,形成大企业顶天立地、小企业铺天盖地、大中小企业协作发展的格局。

产业转型是一个艰苦、持续的过程,也是一项复杂的社会工程,必须创新体制机制,形成鲜明导向,持续加以推进。要强化规划引领。全面落实已经出台的农业转调创、工业提升、服务业提速、园区转型等专项规划,同时不断完善转型发展的“顶层设计”。要完善产业标准的倒逼机制。建立逐步加严的产业标准体系,限期淘汰一批,倒逼提升一批。要形成产业更新机制。形成产业就地更新、自动更新的内在动力。要用好外力。适时邀请国内外专业机构搞好咨询,参与重组。最根本的,是要用足用好市场机制,使企业在资本市场中体现价值,优化重组,实现凤凰涅槃、浴火重生。

(二)以科技创新为重点,在推动创新发展上实现新突破。实现转型发展,最根本的,是要不断提高创新对经济社会发展的贡献度。要坚持目标导向、问题导向、市场导向,着力解决制约我市科技创新能力的关键问题,加快构建更加完善的科技创新体系。要摸清底数,解决好“靠谁创新”的问题。坚持以“我”为主,面向市场、面向全球,开放办科技。对全市各个层面的创新需求进行普查,对可利用的各类创新资源进行系统调查,建好全市科技创新信息库,为各级服务创新、推动创新提供依据。要明确主攻方向,解决好“围绕什么创新”的问题。制定全市科技创新发展规划,明确目标、落实任务、强化措施,力争用五年左右时间,将我市打造成国内一流的创新型城市。要紧紧围绕全市经济社会发展战略推动创新,明确重大政策、重大平台、重大专项和配套措施；紧紧围绕加快培育现代产业体系推动创新,完善体制机制、搭建创新平台、构建创新体系；紧紧围绕满足企业和全社会的创新需求推动创新,健全服务机制,落实服务主体,搞好分类指导和服务。要优化体制机制,解决好“靠什么推动创新”的问题。加快建立健全科技创新市场体系,形成项目选择、成果评价、定价交易、利益分配的企业主体、市场主导机制,分层次构建开放式、市场化的科技资源对接平台。国内重点建立起与中科院等重点院所的深度对接合作机制,境外重点加强与欧美、以色列以及台湾等国家和地区的对接合作。办好全市科技成果交易洽谈会和分产业的专项对接会。筹建市技术交易服务中心,完善技术成果评估和交易体系,加快科技成果资本化步伐。实施孵化器提升计划,按照专业化、企业化、国际化要求,改造提升一批已有的孵化器,有序新规划建设一批专业孵化器,适时规划建设好境外孵化器。大力支持国内外企业(机构),尤其是民营企业创办、领办或参与科技产业园区、科技孵化器及其他科技公共平台建设运营。大力培育引进一批科技咨询、服务、评估、投资、交易等科技类中介机构。要加大资源整合力度,解决好“如何利用好创新资源”的问题。重视发挥中央、省驻潍高校、企业、机构在全市创新发展中的作用。支持发展多种形式的创新联盟,促进院所与院所、院所与企业、市内与市外等全方位的创新资源高效整合。科技只有与金融深度融合,才能实现大的发展。实施科技金融创新计划,促进科技金融业大发展。依托市金融控股集团,成立市科技金融创新服务中心,年内新引进4家以上知名风投机构。各专业孵化器要确保至少有1家风投机构参与服务。要更好地发挥政府职能,解决好“谁来更好服务”的问题。遵循规律,厘清边界,进一步提升政府在规划引导、平台建设、体制创新、人才环境等方面公共服务的能力和水平,解决好一个企业、一个院所办不了、办不好的事情。加大科技投入,创新使用办法,提高财政科技资金使用效率。今年市财政新增2亿元,扩大市科技发展基金。全社会科技研发投入占地区生产总值的比重达到2.27%。实施科技创新“51512”计划:以构筑现代产业体系为主线,新建5处产业科技孵化器,跟踪扶持10个重大科技专项,推动自建或合建50个重点研发机构或创新联盟,新投入运用1000项重点科技成果或孵化成科技型中小企业,新投入使用专业孵化器20万平方米。大力实施人才战略,继续加大高端人才培养引进力度,探索实行国际通用的人才机制和政策。规划建设高层次人才社区,解决好高端人才子女就学等关键问题。探索建立更加有效的知识产权保护机制,打造全国知识产权保护高地。

(三)全面深化改革,在体制机制创新上实现新突破。全面落实中央、省和市委关于深化改革的决策部署,正确、准确、有序、协调推进各领域改革,以改革为统领,推动经济社会全面协调发展。深化农村改革。基本完成农村土地承包经营权确权登记颁证,加快农村集体资产股份制改造。落实集体所有权、稳定农户承包权、放活土地经营权,实现农民土地承包权和经营权分置并行。有序推进农民住房财产抵押、担保、转让。建设齐鲁农村产权交易中心和县市分中心。深化征地制度改革,提高农民在土地增值收益中的分配比例。以土地托管、领办合作社为切入点,深化供销社改革。建立和完善现代企业制度。积极发展混合所有制经济。完善公司法人治理,推行职业经理人制度。深化国有资产管理体制改革,进一步完善各级政府投融资平台治理运营结构。推动经营性文化单位进行公司制、股份制改造。出台支持民营经济发展的政策措施,支持民营企业建立现代企业制度。完善现代市场体系。深化流通体制改革,加快要素市场体系建设。完善主要由市场决定价格的机制,健全居民用水、用电、用气阶梯式价格制度,推进药品、医疗、教育、交通等公用公益事业价格改革。缩小征地范围,规范征地秩序,逐步建立城乡统一的建设用地市场。深化财税改革。加快构建全面规范、公开透明的预算制度。向市人代会报告全口径政府预算,公开市县两级财政总预决算、部门预算和“三公”经费预算。深化预算绩效管理,加快建立“花钱必问效、无效必问责”机制。按照国家统一部署,推进税收制度改革,深入开展“营改增”试点。稳步推进金融改革。鼓励小额贷款公司、村镇银行等中小型金融机构加快发展,稳步推动由民间资本发起设立银行。规范设立民间资本管理机构、民间融资登记服务机构。探索发展介于现货与期货之间的商品交易市场。加快建设中国(潍坊)路演中心。支持大型企业集团设立财务公司。推动企业到多层次资本市场上市挂牌融资。进一步推进金融保险业创新发展。加快支付、结算等金融后台服务中心建设。加快事业单位分类改革。加快建立“管办评分离”的现代教育制度,深入推进学校去行政化改革。逐步取消市属及以下科研院所、医院等单位的行政级别。深化公益性文化事业单位改革。推进机关事业单位养老保险制度改革和民办非营利性机构社会保障制度改革。加快行政管理体制改革。全面完成市县政府机构改革。进一步加大行政审批改革力度,审批事项再压减20%以上,审批效率再提高30%以上。按照“非禁即入”“非禁即准”的原则,鼓励民间资本、国外资本与国有资本平等进入教育、医疗、文化、养老等公共领域和城乡公益设施、重大基础设施建设领域。在基本公共服务、社会管理服务等5个方面推行政府购买服务。深化工商注册管理制度改革,全面推行“先照后证”。加快建立完善政府提供公共服务标准体系。深入推进行业协会、中介组织与行政机关脱钩。创新开发区发展体制,对各类创业服务中心等服务性机构全部推行专业化服务,对各类“区中园”建立市场化、企业化运营模式。将5类公共资源纳入统一平台交易。对我市承担的29项国家级改革试点、11项省级改革试点,要抢抓机遇,逐一制定方案,大胆改革创新,努力创出新路。同时,积极争取更多的改革试点。

(四)深化对外交流合作,在开放发展上实现新突破。创新外贸发展方式。创新办展办会方式,优化出口市场结构、产品结构,新培育10个外贸转型升级示范基地,增设50个海外营销网点,鼓励17个省级出口名牌企业开展国际商标注册、质量标准认证、专利申请,支持有条件的企业创建、并购国际品牌。建成跨境电子商务平台。积极参与丝绸之路经济带和海上丝绸之路建设。支持企业进口转型发展所需设备、技术以及大宗资源类产品。创新招商方式。建立完善合作发展促进机制,构建全方位对外合作格局。探索完善招商模式“两个转型”、“四位一体”利用外资、全方位引进境外优质资源的具体政策措施。细分市场、细分责任,形成职业化对接服务机制,力争引进市外各类机构300家以上、资金1000亿元以上。创新经济合作方式。支持有条件的企业全球布局产业链,开展境外资源开发、企业并购重组、人才引进和国际研发合作。支持综合保税区加快对接国际自贸区规则,争取开展有特色的自由贸易试点。推动中日韩地方经济合作示范区建设取得实质成果,办好中日韩博览会。深化与德国弗莱辛地区城镇化伙伴关系。务实办好国际风筝会和鲁台经贸洽谈会。

(五)加快重点区域开发建设,在统筹区域发展上实现新突破。大力实施“蓝黄”战略。举全市之力突破滨海。突出海洋特色,抓好港口带动,加快建设海洋经济新区和现代工业新城。创新政府服务,探索试行负面清单、权力清单、服务清单模式。大力发展海洋运输、临港物流、海工装备、生物医药、海洋化工等海洋特色产业,加快形成海洋产业聚集区。在加快推进白浪河、弥河等河流治理的基础上,大规模开展沿路、沿河、沿海和重点片区绿化,严格项目准入和环评标准,大力度提升生态建设水平。启动建设科技大学起步区和山东职教公共实训基地,13所在建院校部分建成并达到招生条件。中央商务区一期、旅游度假区海洋公园、游客接待中心投入使用,9个生态小镇全部开工、2个建成。加快推进综合保税区B区建设。大力提升市区。坚持国际视野、一流标准、创新驱动、一体发展,力争用5年左右的时间,将市区打造成全市创新发展的引擎、新兴高端产业的高地和服务全市、辐射周边的中心。制定实施市区产业发展规划,建立产业更新机制,促进城市产业发展与设施建设、服务功能协调发展。实施市区产业发展“3·10”工程,突出抓好10大产业、10个重点园区、10条特色街区。按照创新、智慧、绿色、人文、畅通的理念,实施一批提升市区功能品质重大专项。全面启动全国循环经济示范市建设和中美低碳生态城市试点,实施“211绿色低碳建筑行动”。加快中国食品谷、齐鲁台湾城等重点片区开发建设。实施市区畅通工程,对部分主次干道、片区实施微循环改造；完成北环路东西延伸段、潍县路、潍州路和潍昌路改造工程,开工建设宝通街东延项目。深化行政体制改革,制定市区各级权责清单,理顺市区各级财权事权关系,放活市场、放活基层、放活政府公共资源,提升城市效率和发展活力。实施“品牌潍坊”战略,大力培育产品品牌、企业品牌、服务品牌、城市品牌。加快开发两河。对潍河、弥河实施全流域规划、保护和开发,通过10年左右努力,把“两河”流域打造成为功能完善的生态带、经济转型升级的隆起带、农业现代化与城镇化互动发展的示范带。今年,制定好规划和推进计划,启动河道生态治理、交通设施建设等基础工作。统筹重大基础设施建设。潍坊港2万吨级航道实现通航、5万吨级航道加快建设；潍日高速全面开工；机场迁建编制完成预可研报告。按照省统一部署,全力做好高铁枢纽、济青中线前期工作。疏港铁路项目完成设计和开工手续。鲁辽陆海货滚甩挂运输通道开工建设。全面启动“智慧潍坊”行动计划,抓好电子政务、智能交通、智慧社区等十大工程。

(六)以人的城镇化为核心,在加快构建新型城镇化体系上实现新突破。推进城乡建设由扩张型向内涵式发展转型。突出以人为本。既注重统一规划引导,又充分尊重群众意愿,满足人的需求,坚决避免不切实际的一刀切和赶农民上楼等现象。深化城乡户籍一体化改革,出台促进城镇常住人口有序市民化的政策措施,推进教育、医疗、社保等基本公共服务覆盖城镇常住人口。完善农民非农就业免费培训政策。强化规划引领。坚持规划先进、规划先行。瞄准打造百年城镇目标,结合历史文化、田园风光等因素,出台覆盖城乡的城镇化发展规划,编制完善各专项规划,形成引领新型城镇化的规划体系。坚持产城一体。以工业化引领城镇化,走“两化”同步、“两区”同建、融合发展的路子。精心培育一批工业强镇、商贸重镇、旅游名镇、特色小镇。着力完善城镇功能,提升承载能力。支持有条件的农村社区同步规划发展特色产业园区,促进农民就地转移就业。实施城乡社区提升工程,合理解决社区建设中的关键问题,增强社区服务功能。年内新开工建设农村新型社区147个,建成100个。创新体制机制。全面落实扩权强镇政策措施,适时扩大试点范围。制定推动城市公共服务进镇村、鼓励各类市场主体下乡镇的具体措施。大力开展小城市、特色小城镇试点,加快提升16个省级示范镇和全国重点镇建设水平。推动非农产业主导的规模乡村建设新型城镇化社区,人口规模达到镇标准的按镇管理,达到城市标准的按城市管理。推进协调发展。突出生态优先、城乡一体。促进中心城市、县城、小城市和小城镇、农村社区自然天成、功能互补、协同发展。按照生产空间集约高效、生活空间宜居适度、生态空间山清水秀的要求,促进生产生活生态协调发展。

(七)以保障和改善民生为重点,在加强社会建设上实现新突破。继续加大力度改善民生,全市民生支出占公共财政支出的比重达到60%以上。坚持普惠性和重点救助相结合,今年继续办好7个方面25件民生实事(附后)。进一步提升创业就业服务和社会保障水平。实施全民创业计划,进一步降低门槛、放宽领域、加大支持力度,设立创业专项资金,筹建潍坊创业大学,完善创业辅导体系,加快建设创业城市。每个县市区设立1处农民工服务中心。健全完善“兜底线、保基本、可持续”的社会保障机制,整合城乡居民基本医疗保险制度。加大扶贫和社会救助工作力度,更好地解决困难群众的生产生活问题,确保“人人过得去”,力争“人人过得好”。繁荣教育、卫生、文化等社会事业。实施新一轮农村义务教育学校校舍标准化建设工程、市区中小学标准化建设提升工程,用三年左右时间,解决市区大班额问题。鼓励发展民办教育,支持知名团队办教育,满足多层次教育需求。重视支持高等教育和职业教育。加快建设城乡健康管理体系,为群众提供优良的健康服务。促进体育事业健康发展。落实社会主义核心价值体系建设的具体措施,不断激发全社会向上的力量、向善的力量。探索建立社会征信体系,推进社会诚信建设制度化。充分挖掘民间收藏资源,发展好博物馆事业。完善现代公共文化服务体系,广泛开展群众性精神文明创建活动,争创全国文明城市。进一步改善城乡群众基本生活条件。继续扩建、新建农村公路,新建设一批公共租赁住房,再改造一批城市老旧小区。继续增加市区绿色公交车和公共自行车数量。提高社会治理水平。深入推进平安潍坊、法治潍坊建设,加强社会治安综合治理。依法严厉整治和规范建筑市场秩序,严肃查处拖欠农民工工资等违法违规问题。加强社区“三支队伍”建设,解决社区运行中的突出问题。做好拥军优属、国防动员、人民防空、民兵预备役工作。促进计划生育、妇女儿童、民族宗教、对口支援等健康发展。

各位代表,安全发展关系到人民群众的生命财产安全；生态环境事关人民群众的身体健康和生活质量,是科学发展必须严守的两条“底线”:一要确保安全发展。始终把安全摆到“高于一切、先于一切、重于一切”的位置,凡是与安全发展矛盾的,服从安全；凡是安全发展需要的,优先安排。全面开展安全生产标准化规范化建设,织密织牢基层安全保障网。实施更加严格的隐患排查整改制度,从源头上消除安全隐患。加大投入,配齐配强各级消防安全装备；规划建设好各级防灾减灾中心,开展全民防灾减灾教育训练,增强防灾减灾能力。全面落实党政同责、一岗双责、分级负责、齐抓共管的责任体系。同时,要采取得力措施,统筹管控好金融安全、政府债务安全、舆情安全和社会安全。二要确保生态环境质量。坚持生态优先,走绿色发展道路。宁肯发展的速度慢一点,也要把生态环境保护好、治理好。要强化目标管控。力争用三年左右时间,使全市排放总量、环境质量主要指标与全市经济总量相适应。全面落实“386”环保行动计划,坚决打好消灭污水直排、自备井整治、河道生态修复、煤改气、空气异味整治、淘汰黄标车、防控城市扬尘污染、治理农业面源污染等八大攻坚战,全年空气污染物浓度下降6%,23条重点河流水质改善率再提高15%以上。全面落实国家循环经济试点市工作方案,大力推动产业、交通、建筑和市民生活低碳化、排放减量化。全市碳排放和排污权交易机构上半年投入运行。划定并严守土地红线、生态红线。规划建设城市森林公园和生态隔离带。积极创建国家森林城市。大力推行城乡环卫一体化、垃圾资源化利用。实行逐步加严的环境准入和管理标准,建立健全分区环境指标定期发布制度、社会监督制度、区域限批制度和严格的问责制度。对破坏生态环境的各类案件和环境监管失职渎职行为,发现一起,查处一起,曝光一起!

三、加强政府自身建设

适应市场化、信息化、国际化的新形势和转型发展的新要求,顺应人民群众的新期待,按照建设“五型”政府的目标,大力加强政府自身建设。

(一)大力加强作风建设。扎实开展第二批群众路线教育实践活动,对照为民务实清廉主题,认真查摆、深入整改在“四风”方面存在的突出问题。强化职业意识,铭记职业责任,做克己奉公、为民务实的公务员。用心做事、用心履职,真正“深下去”,及时了解民情,倾听呼声,回应群众关切；适时“跟上去”,争取支持,赢得指导；善于“走出去”,学习经验,把握动态,推动合作；潜心“钻进去”,深入调研,超前谋划,寻求良策。进一步强化问题导向、基层导向,逐级建立问题专报和协调解决机制。建立完善政府智库。规范政府顾问、专家论证、专项评估等决策制度。

(二)提高行政效能。开展创建效能机关、效能岗位活动,用开放的视野和竞争的理念认识效能、评判效能、提升效能。瞄准国内外先进标杆,找差距,寻对策,上水平,创一流。要创新体制机制提高效能。市直各部门单位都要制定并落实好改革创新计划。

对影响效能的行政审批、绩效管理、电子政务等关键问题,都要运用改革的办法,逐一研究对策,加以解决。

(三)坚持依法行政。要心存敬畏,敬重法纪,恪守规矩,依法依规办事。严格执行人大及其常委会的决议决定,认真办理人大代表议案建议和政协委员提案,重视发挥各民主党派、工商联、社会各界以及工会、共青团、妇联等人民团体的作用。出台《潍坊市法治政府建设规划纲要》。发布政府部门权责清单,完善权力运行监督制约机制。继续完善规范性文件、对外合同协议合法性、合理性审查制度。深入推进政务公开,规范建立法律顾问制度,提高政府法治化水平。

(四)努力提升职业化水平。引导广大公务员人人争做行家里手,事事做成精品工程。开展公务员职业化建设制度试点,探索建立以职业化为目标的机关事业单位岗位分类管理制度。根据转型发展要求,制定公务员职业素质建设指导意见。今年重点抓好金融、商务、应急等知识和技能培训。

(五)坚持廉洁从政。自觉贯彻落实中央八项规定,严格规范各种职务行为。厉行勤俭节约,今年再压减市直部门一般性经费预算5%。加快推进惩治和预防腐败体系建设,努力从源头上杜绝各种腐败案件的发生。严格落实党风廉政建设责任制,完善和规范行政监察、经济审计等制度,及时查处各种违纪违法案件,努力做到干部清正、政府清廉。

各位代表,新的一年,新的任务；新的机遇,新的希望。让我们紧密团结在以习近平同志为总书记的党中央周围,在中共潍坊市委的坚强领导下,凝心聚力、开拓进取、创新发展,奋力开创潍坊科学发展、率先发展的新局面!
